# Measurement of myocardial native T1 in cardiovascular diseases and norm in 1291 subjects

**DOI:** 10.1186/s12968-017-0386-y

**Published:** 2017-09-28

**Authors:** Joanna M. Liu, Alexander Liu, Joana Leal, Fiona McMillan, Jane Francis, Andreas Greiser, Oliver J. Rider, Saul Myerson, Stefan Neubauer, Vanessa M. Ferreira, Stefan K. Piechnik

**Affiliations:** 1 Oxford Centre for Clinical Magnetic Resonance Research (OCMR), Division of Cardiovascular Medicine, Radcliffe Department of Medicine, University of Oxford, Oxford, OX3 9DU UK; 2 Siemens Healthineers, Erlangen, Germany

**Keywords:** Cardiac magnetic resonance, T1-Mapping, ShMOLLI, Reference myocardium, Affected myocardium, Late gadolinium enhancement

## Abstract

**Background:**

Native T1-mapping provides quantitative myocardial tissue characterization for cardiovascular diseases (CVD), without the need for gadolinium. However, its translation into clinical practice is hindered by differences between techniques and the lack of established reference values. We provide typical myocardial T1-ranges for 18 commonly encountered CVDs using a single T1-mapping technique – Shortened Look-Locker Inversion Recovery (ShMOLLI), also used in the large UK Biobank and Hypertrophic Cardiomyopathy Registry study.

**Methods:**

We analyzed 1291 subjects who underwent CMR (1.5-Tesla, MAGNETOM-Avanto, Siemens Healthcare, Erlangen, Germany) between 2009 and 2016, who had a single CVD diagnosis, with mid-ventricular T1-map assessment. A region of interest (ROI) was placed on native T1-maps in the “most-affected myocardium”, characterized by the presence of late gadolinium enhancement (LGE), or regional wall motion abnormalities (RWMA) on cines. Another ROI was placed in the “reference myocardium” as far as possible from LGE/RWMA, and in the septum if no focal abnormality was present. To further define normality, we included native T1 of healthy subjects from an existing dataset after sub-endocardial pixel-erosions.

**Results:**

Native T1 of patients with normal CMR (938 ± 21 ms) was similar compared to healthy subjects (941 ± 23 ms). Across all patient groups (57 ± 19 yrs., 65% males), focally affected myocardium had significantly different T1 value compared to reference myocardium (all *p* < 0.001). In the affected myocardium, cardiac amyloidosis (1119 ± 61 ms) had the highest native T1 compared to normal and all other CVDs, while iron-overload (795 ± 58 ms) and Anderson-Fabry disease (863 ± 23 ms) had the lowest native reference T1 (all p < 0.001). Future studies designed to detect the large T1 differences between affected and reference myocardium are estimated to require small sample-sizes (*n* < 50). However, studies designed to detect the small T1 differences between reference myocardium in CVDs and healthy controls can require several thousand of subjects.

**Conclusions:**

We provide typical T1-ranges for common clinical cardiac conditions in the largest cohort to-date, using ShMOLLI T1-mapping at 1.5 T. Sample-size calculations from this study may be useful for the design of future studies and trials that use T1-mapping as an endpoint.

**Electronic supplementary material:**

The online version of this article (doi:10.1186/s12968-017-0386-y) contains supplementary material, which is available to authorized users.

## Background

Cardiovascular magnetic resonance (CMR) offers a range of methods for non-invasive myocardial tissue characterization in cardiovascular diseases [1–4]. Late gadolinium enhancement (LGE) enables accurate delineation of the size and location of myocardial infarctions [3], and the pattern of non-ischemic LGE has diagnostic value for cardiomyopathies [5–7]. However, signal intensities on LGE imaging are displayed on relative grayscales, rendering clinical interpretation subjective, depending on threshold-windowing and the quality of nulling of the “reference” myocardium, which may not be present in diseases with global involvement or diffuse interstitial fibrosis [8–11]. From a safety viewpoint, LGE requires administration of gadolinium-based contrast agents (GBCA), which are contra-indicated in patients with end-stage renal failure due to risks of nephrogenic sclerosing fibrosis [12]; more recently, reports also suggest that GBCA can deposit in the brain, especially with repeated MRI scans and accumulative GBCA exposure [13–15].

Native T1-mapping is a quantitative and GBCA-free myocardial tissue characterization method that can detect changes in a variety of cardiac conditions, sometimes beyond what LGE imaging can reveal [16, 17]. T1 (proton spin-lattice relaxation time) is a CMR property of tissue, prolonged by increased free water content [18, 19]. Each tissue type, including the myocardium, has its own normal range of T1 values, deviation from which may be indicative of disease [18]. Native T1-mapping has widely proven sensitivity to pathological changes in diseases, including detection of myocardial edema, infarction, ischemia, cardiomyopathies and diffuse fibrosis [2, 6–8, 11, 20–29].

Currently, the application of T1-mapping for the clinical diagnosis of cardiovascular diseases is hindered by a lack of standardization for the methods used and the different reference T1 values. A wide range of different T1-mapping techniques, each with different normal T1 ranges, are being used to study relatively small (*n* < 100), typically highly pre-selected patient cohorts, often without accounting for regional variations in myocardial pathology [20, 22, 24, 30]. Hence, despite its obvious advantages, native T1-mapping has yet to make full translation from proof-of-principle studies to wide adoption in clinical practice, which requires a stable method with significant published clinical evidence. Establishing reference T1 values for normal and disease conditions using a single stable T1-mapping method is pivotal towards widespread clinical applications, and to provide reliable sample size calculations to guide the design of future studies and clinical trials.

T1-mapping using the Shortened Modified Look-Locker Inversion Recovery (ShMOLLI) technique has been validated in single- and multi-center clinical studies for a variety of cardiovascular diseases [17–28, 30–41]. It is also used in the UK Biobank (over 10,000 datasets acquired; projected total: 100′000, [42, 43]), and the ongoing multi-centre Hypertrophic Cardiomyopathy Registry study (HCMR; 2750 patients, [42–44]). We have a large resource of clinical and research scans with T1-mapping accumulated from pooled evidence from the past 7 years [18, 19, 23, 24, 26, 28, 30, 31, 34, 35, 39, 45]. In this study of 1291 subjects, we characterized commonly encountered clinical myocardial conditions using T1-mapping, derived native T1 ranges, and produced sample-size calculations to guide future clinical studies and trials.

## Methods

### Study population

To study myocardial T1 values of cardiovascular diseases, we screened CMR scans undertaken for relevant scans, clinical and research, performed between June 2009 and June 2016 in our tertiary-referral CMR unit, the Oxford Centre for Clinical Magnetic Resonance Research (OCMR), Oxford, United Kingdom. We included the scans of patients diagnosed with a single cardiovascular disease and patients with normal CMR (no cardiovascular disease or significant comorbidities and normal ECG), as determined by at least one clinical consultant cardiologist CMR expert. We excluded scans without T1-maps, in patients under 18 years old, repeated in the same patient and in patients diagnosed with more than one cardiovascular disease (e.g. hypertrophic cardiomyopathy and coronary artery disease). We also excluded patients (*n* = 53) who had a history of cardiovascular risk factors (e.g. diabetes mellitus or smoking) and/or abnormal ECG, but had no clear-cut features of cardiovascular disease on CMR (Additional file 1: Table S1). We included only good-quality T1-maps with good R^2^ goodness-of-fit maps, free from artefacts on inversion recovery (IR)-weighted images, as previously published [24]. Following the peer review, we performed an additional 4 month (July 2016 – December 2016) targeted search for three diseases underrepresented in the original sample (iron overload, Anderson Fabry Disease and atrial fibrillation).

In total, 1291 CMR scans were included in final analysis and all scans had a mid-ventricular short-axis T1-map, defined by the clear presence of LV papillary muscles and located approximately half-way between the mitral valve annulus and the LV apex on long-axis cines [46]. The study comprised clinical scans of patients with a single cardiovascular disease (*n* = 1221), clinical and re-analysis of our prior research scans for patients with pheochromocytoma [23], Takotsubo cardiomyopathy [24], and acute myocarditis [25]), and clinical scans of patients found to have normal CMR (*n* = 70, no cardiovascular history and normal ECG, Additional file 2: Table S2).

This study was approved by the relevant local ethics authorities. All study subjects gave written informed consent.

### CMR protocol

All CMR scans were performed at 1.5 T (Magnetom Avanto, Siemens Healthcare, Erlangen, Germany) using established techniques as previously described [25]. These included long- and short-axis cines, LGE, and native T1-mapping (ShMOLLI prototype sequence with inline map generation, WIP 561 and 448C) [37]. Precise statement on the WIP prototype used is essential to highlight that ShMOLLI underwent only one change in the inversion time calculation required to address regulatory issues, with clearly documented impact on the measured T1 values [37].

### T1-Mapping analysis

Separate data files containing all T1-maps were created and anonymized before analysis by an observer blinded to clinical information. To assess the impact of focal pathology on native T1 changes, regions of interest (ROIs) were manually placed on mid-ventricular T1-maps in areas corresponding to focal enhancement on LGE images. In diseases without focal LGE, ROIs were placed as follows. For Takotsubo cardiomyopathy, ROIs were placed in areas on T1-map corresponding to the maximal regional wall motion abnormality (RWMA) [24], defined as severe hypokinesia, akinesia or dyskinesia on short-axis cines. For iron overload and Anderson Fabry disease, the ROIs were placed in the septum of the mid ventricular slice [28]. For AL amyloid which did not have LGE negative myocardium, a single septal ROI was drawn. Where possible, ROIs were drawn as large as possible to span one-sixth of the myocardial circumference, with particular care taken to avoid partial-volume contamination from the surrounding blood pool. A reference ROI was placed in myocardium as far as possible from any focal changes. In the 70 patients with normal CMR and normal ECG, a ROI was placed in the septum. The mean T1 values within the ROI were reported with 1SD.

All T1 values presented in this study apply to the contemporary WIP448C, 780B and 1048. A significant proportion of T1 values in this study (42% or 541/1273 of the subjects) was measured using the original ShMOLLI WIP561 [18, 19]. The small known difference between this and all subsequent distributions arising from the change in inversion time calculations was compensated for with an empirical formula T1_(all current WIPs)_ = 1.0221*T1_(WIP561)_ - 32.795, established with material previously described [37]. Overall, these corrections caused a trend towards lower mean native T1 values (by ~10 ms) without reaching statistical significance: these changes are within the variability (2%) of the method [18, 19]. For consistency, we performed the same T1 correction on our previously published normal T1 values in 342 healthy volunteers [19], which reduced the mean normal native T1 by 12 ms (uncorrected normal T1 953 ± 23 ms using eroded myocardial contours vs. corrected eroded normal T1 941 ± 23 ms). Consequently, 941 ± 23 ms was used as the healthy normal T1 for this study and for sample size calculations.

### Statistical analysis

All data are parametric, as determined by the Kolmogorov-Smirnov test, and were expressed as mean ± SD. Paired samples were assessed by paired Student *t*-test and unpaired samples were assessed by the unpaired 2-tailed Student *t*-test. Comparisons between ≥3 separate data groups were performed using analysis of variance (ANOVA) with Bonferroni post-hoc correction. The associations between native T1 values, clinical demographics and LV function were analyzed using multi-variable analysis with stepwise selection method for significant associations (*p* < 0.05). All data were analyzed on per-subject basis using MedCalc 12.7.8 (Ostend, Belgium). In all cases, p < 0.05 denotes statistical significance.

### Sample size calculations for future studies using ShMOLLI native T1-mapping

Calculations of sample sizes and effect sizes (Cohen’s d) were performed for two comparisons, using methods as previously described [47–49]: (1) for detecting significant differences between native T1 in the most affected myocardium (LGE+, or RWMA+ for Takotsubo cardiomyopathy) and reference myocardium in patients; and (2) for detecting significant differences between native T1 of reference myocardium in patients (LGE-, or RWMA- for Takotsubo cardiomyopathy) and T1 of healthy volunteers (2-pixel eroded and WIP_442_-corrected; normal ShMOLLI T1 = 941 ± 23 ms) [19].

In brief, the sample size required for native T1 values to show a significant change with power of 80% and α error of 0.05 was calculated using the following formula [47, 49]:

$$ n\kern0.5em =\kern0.5em f\left(a,P\right)\ast {\sigma}^2\ast \frac{2}{\delta^2} $$

Where n is the sample size needed, α is the significance level, *P* is the study power, and *f* is the value of the factor for different values of α and *P*, with σ as the inter-study standard deviation and δ as the difference to be detected [49]. The effect size (Cohen’s d) was also determined as the differences between means divided by the pooled SD for each comparison, as previously described [48].

## Results

### Subject characteristics

Subject characteristics are summarized in Table 1. CMR scans of 1291 patients (57 ± 19 yrs., 65% males) were analyzed, which included 16 different cardiovascular diseases and a group of patients with normal CMR. Patients with normal CMR were used as the study controls, with an important distinction from previously published healthy volunteers [19], which was also used to define normality for sample size calculations.

**Table 1:** Characteristics of study subjects

	n	Age (years)	Male (n)	BMI (kg/m^2^)	HR (bpm)	LVEF (%)	LV mass index (g/m^2^)	LV Wall_min_ (mm)	LV Wall_max_ (mm)
Patients with normal CMR	70	48 ± 17	45	24 ± 3	65 ± 13	66 ± 9	56 ± 14	7 ± 1	10 ± 2
Cardiac Amyloidosis (AL)	32	73 ± 11	11	28 ± 5	73 ± 13	55 ± 15^a^	111 ± 32	12 ± 4	18 ± 3^a^
Cardiac Amyloidosis (ATTR)	22	75 ± 13	14	26 ± 4	79 ± 19	53 ± 14	120 ± 16	12 ± 3	20 ± 5
Anderson-Fabry disease^b^	21	50 ± 17	20	27 ± 5	62 ± 14	60 ± 15	62 ± 25	8 ± 2	13 ± 5
Aortic Stenosis	24	63 ± 18	19	29 ± 7	73 ± 13	62 ± 13	83 ± 23	9 ± 3	14 ± 3
Atrial Fibrillation^b^	23	66 ± 11	18	28 ± 4	89 ± 19	50 ± 13^a^	61 ± 13	7 ± 1	11 ± 2
Chronic CAD	309	62 ± 12	193	28 ± 5	69 ± 14	50 ± 16^a^	74 ± 23	7 ± 2	12 ± 3
Dilated Cardiomyopathy	151	60 ± 15	98	27 ± 7	72 ± 15	35 ± 14^a^	81 ± 29	5 ± 2^a^	8 ± 2
Hypertrophic Cardiomyopathy	185	56 ± 15	138	29 ± 11	68 ± 12	69 ± 12	88 ± 33	9 ± 3	19 ± 4^a^
Hypertension	59	62 ± 14	42	29 ± 5	68 ± 15	61 ± 16	83 ± 38	9 ± 2	14 ± 3
Cardiac Iron-Overload^b^	23	53 ± 21	17	23 ± 9	77 ± 14	65 ± 13	56 ± 27	7 ± 2	14 ± 3
Myocarditis (acute)	146	41 ± 12^a^	68	27 ± 5	75 ± 13	58 ± 13	69 ± 18	8 ± 2	12 ± 2
Myocarditis (previous)	93	47 ± 17	72	27 ± 4	69 ± 14	61 ± 10	62 ± 17	7 ± 1	11 ± 2
Obesity	38	53 ± 15	22	35 ± 4^a^	70 ± 14	60 ± 10	60 ± 21	7 ± 1	10 ± 2
Pheochromocytoma	29	50 ± 14	14	25 ± 6	71 ± 29	65 ± 10	57 ± 12	8 ± 1	10 ± 1
Cardiac Sarcoidosis	21	59 ± 9	10	28 ± 6	74 ± 13	60 ± 14	64 ± 20	8 ± 2	13 ± 3
Takotsubo cardiomyopathy	45	64 ± 12	35	25 ± 5	74 ± 18	60 ± 15	58 ± 17	8 ± 1	11 ± 3

All values are n (%) or mean ± SD. *ARVC* arrhythmogenic right ventricular cardiomyopathy, *BPM* beats per minute, *BMI* body mass index, *CAD* coronary artery disease, *g* gram, *HR* heart rate, *kg* kilograms, *LVEF* left ventricular ejection fraction, *LV* left ventricular, *m* metre, *mm* millimetre, *Max* maximum, *Min* minimum

^a^denotes values significantly different from patients with normal CMR (all *p* < 0.05)

^b^indicates material from extended analysis period included to address peer review

Compared to patients with normal CMR, patients with acute myocarditis were statistically younger (all *p* < 0.05); patients with obesity had higher body mass index; patients with dilated cardiomyopathy, cardiac amyloidosis and chronic coronary artery disease had significantly lower LV ejection fraction (all p < 0.05); patients with hypertrophic cardiomyopathy and cardiac amyloidosis had significantly higher maximal LV wall thickness (all p < 0.05), while patients with dilated cardiomyopathy had a significantly lower minimum LV wall thickness (all p < 0.05).

### Normative native T1 ranges in cardiovascular disease and norm

Native T1 in typical tissue classes in cardiovascular diseases and in patients with normal CMR are shown in Table 2. In this study, gender did not affect myocardial T1 values significantly (2-way ANOVA, data not shown), and thus only overall T1 values are provided. Patients with normal CMR (938 ± 21 ms) had similar native T1 compared to healthy volunteers [19] (941 ± 23 ms).

**Table 2:** Normative ranges for the native ShMOLLI-T1 ranges for the most common myocardial tissue conditions encountered in clinical practice

Native T1 [ms]	Reference myocardium	LGE+ or RWMA+ myocardium
Patients with normal CMR	938 ± 21	–
Cardiac Amyloidosis (AL)	–	1158 ± 75
Cardiac Amyloidosis (ATTR)	1002 ± 63	1061 ± 29
Anderson-Fabry Disease^c^	863 ± 23	902 ± 17
Aortic Stenosis	952 ± 20	1019 ± 23^a^
Atrial Fibrillation^c^	945 ± 25	1010 ± 54
Chronic CAD	951 ± 33	1078 ± 94^a^
Dilated Cardiomyopathy	945 ± 27	1038 ± 38^a^
Hypertrophic Cardiomyopathy	932 ± 81	1041 ± 86^a^
Hypertension	944 ± 24	1022 ± 43^a^
Cardiac Iron-Overload^c^	795 ± 58	–
Myocarditis (acute)	947 ± 39	1058 ± 74^a^
Myocarditis (previous)	941 ± 36	1026 ± 47^a^
Musculo-dystrophy	935 ± 23	1006 ± 10^a^
Obesity	936 ± 22	1031 ± 28^a^
Pheochromocytoma	939 ± 24	1006 ± 20^a^
Cardiac Sarcoidosis	934 ± 47	1030 ± 53^a^
Takotsubo Cardiomyopathy^b^	988 ± 41	1093 ± 64^a^

All values are mean ± SD. *RWMA* regional wall motion abnormalities, *LGE* late gadolinium enhancement. All other abbreviations are as per Table 1

^a^p < 0.001 compared to native T1 of reference myocardium

^b^Disease entity in which affected myocardium is characterized by regional wall motion abnormalities (RWMA) only

^c^- indicates material from extended analysis period included to address peer-review

All cardiovascular diseases demonstrated focal enhancement on LGE imaging, except for Takotsubo cardiomyopathy and iron overload, where no LGE was detected, and regional wall motion abnormality (RWMA) was used to define abnormality. In all diseases with focal LGE, enhanced myocardium had significantly higher native T1 values compared to the unenhanced reference myocardium within the same disease type, all paired *p* < 0.001. For Takotsubo cardiomyopathy, myocardium with RMWA had significantly higher native T1 compared to the reference myocardium without RWMA, paired p < 0.001. There was no LGE negative myocardium in the AL amyloidosis subgroup. AL amyloidosis also had significantly higher T1 values in the affected (LGE positive) myocardium compared to ATTR amyloidosis (1158 ± 75 ms vs 1061 ± 29 ms, respectively, *p* < 0.01), similar to previously published values [50] With respect to “apparently normal” reference myocardium in various cardiac diseases, native T1-mapping was still able to detect additional abnormalities. Despite apparent lack of abnormalities either on cines (Takotsubo cardiomyopathy; RWMA-, 988 ± 41 ms) or LGE (LGE negative reference myocardium in ATTR amyloidosis; 1002 ± 63 ms) the native T1 values were significantly higher compared to patients with normal CMR and the reference myocardium in all other cardiovascular diseases (all *p* < 0.01). In diseases with generally low T1 values, reference myocardium in patients with cardiac iron-overload (LGE-, 793 ± 58 ms) and Anderson-Fabry disease (LGE-, 863 ± 23 ms) had significantly lower native T1 compared to patients with normal CMR and the reference myocardium in all other cardiovascular diseases (all p < 0.01). Further, reference myocardium in patients with cardiac iron-overload had lower native T1 compared to Anderson-Fabry disease (p < 0.01). The above findings support that these diseases are likely global in nature, with myocardial abnormalities beyond LGE detection. In all other cardiovascular diseases, the reference myocardial T1 was comparable with patients with normal CMR and each other (*p* > 0.07 by ANOVA with Bonferroni post-hoc method).

**Fig. 1:**
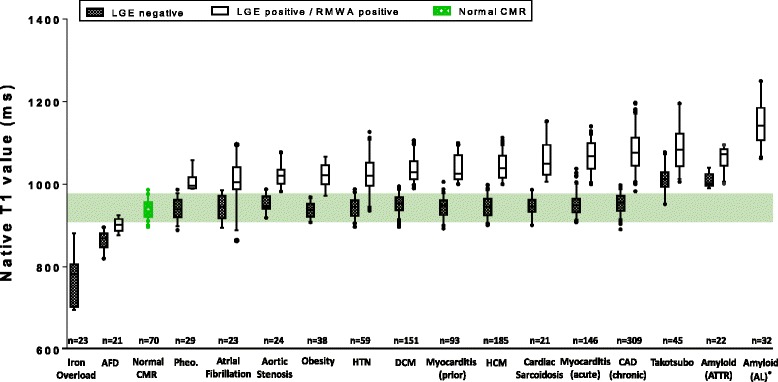
Characteristic native myocardial T1 values (1.5 Tesla) for 16 different cardiovascular conditions, stratified by the presence of late gadolinium enhancement (LGE) or regional wall motion abnormality (RWMA). Data presented as box and whisker plots with the median, upper and lower quartiles, min and max excluding outliers, and outliers that are more than 3/2 the upper and lower quartiles. Disease names are as per abbreviations list. Areas of abnormality for all diseases except Takotsubo cardiomyopathy were defined using LGE, whereby LGE positive denotes myocardial regions with enhancement on LGE images and LGE negative denotes myocardial regions with no enhancement on LGE images. In Takotsubo cardiomyopathy, where there is no enhancement on LGE images, abnormality was defined by the presence of RWMA (RWMA positive). ^*^There were no LGE negative regions in AL Amyloidosis subjects

All data were normally distributed, as determined using the Kolmogorow-Smirnoff test (previously described in statistical methods section), and were presented accordingly in Table 2 as mean ± 1SD. After the peer-review process, the data were also presented in a non-parametric fashion in Fig. 1, to offer a more comprehensive description of the data distribution.

### Sample size calculations

The minimum estimated sample sizes for studies using ShMOLLI native T1-mapping with the typical power calculation assumptions are presented in Table 3. Within-subject differences between the reference myocardium (LGE-, or RWMA- in Takotsubo cardiomyopathy) and focal abnormalities (LGE+, or RWMA+ in Takotsubo cardiomyopathy) are characterized by large effect sizes and relatively small sample size requirements. Conversely, departures of T1 in the reference myocardium in various cardiovascular diseases from healthy-volunteer native T1 [19] are smaller, leading to smaller effect sizes and large sample size requirements. These included diseases such as pheochromocytoma where the effect sizes for comparisons against healthy subjects are small (Cohen’s d = 0.09), similar to that between patients with normal CMR and healthy subjects (Cohen’s d = 0.14), if only using a mid-ventricular T1-map with select manual ROIs without more advanced image analysis. This leads to large sample size requirements for future studies, in the order of a few thousand subjects. Diseases such as cardiac iron-overload, cardiac amyloidosis and Takotsubo cardiomyopathy have large effect sizes for comparisons against healthy volunteers, and offer easily manageable sample sizes of <50 per group for future studies.

**Table 3:** Sample size calculation using native ShMOLLI T1-mapping for clinical studies and trials, arranged according to Cohen’s d effect size (largest to smallest)

	Departure of focally abnormal myocardium from reference myocardium (within subjects)		Departure of reference myocardium from healthy myocardium [19] (between groups)	
	Cohen-d	Paired, n>	Cohen-d	Unpaired, n>
Patients with normal CMR	N/A	N/A	0.14	1604
Cardiac Amyloidosis (AL)	4.58	2	–	–
Cardiac Amyloidosis (ATTR)	3.91	4	1.28	9
Aortic Stenosis	3.39	6	0.68	146
Takotsubo Cardiomyopathy	3.33	6	1.06	32
Dilated Cardiomyopathy	3.09	6	0.56	104
Pheochromocytoma	3.02	6	0.09	3880
Myocarditis (acute)	2.92	6	0.52	120
Obesity	2.81	8	0.21	716
Hypertension	2.36	8	0.57	100
Myocarditis(previous)	2.30	10	0.0	N/A
Cardiac Sarcoidosis	2.28	10	0.0	N/A
Cardiac Iron-Overload^a^	2.06	10	13.30	4
Chronic CAD	2.06	10	0.47	146
Hypertrophic Cardiomyopathy	1.59	16	0.15	1398
Atrial Fibrillation^a^	1.47	18	0.29	376
Anderson-Fabry Disease^a^	0.82	50	2.81	8

All abbreviations are as per Tables 1 and 2. Focally abnormal myocardium: myocardium affected by either late gadolinium enhancement (LGE) or by regional wall motion abnormalities (RWMA) defined as severe hypokinesia, akinesia or dyskinesia on cines in patients with Takotsubo cardiomyopathy. Reference myocardium: myocardium not affected by RMWA or LGE. ^a^indicates material from extended analysis period included to address peer review

## Discussion

This is the first large-scale study with over 1200 subjects to address the need for published normative ranges for myocardial native T1 in a wide variety of cardiac conditions, with a single T1-mapping technique at the most common for clinical CMR 1.5 T field strength.

### Native T1 in patients with normal CMR and healthy subjects

As study controls, we measured the mean native T1 using mid-ventricular septal ROI in patients with normal CMR (no cardiovascular disease with normal ECG, Fig. 1). To further define normal T1 values, we quoted data for the 342 healthy subjects previously used to establish the normal ranges at 1.5 T [19]. Patients with normal CMR had similar native myocardial T1 compared to healthy-subjects T1 [19], which provided a robust baseline for comparison against native T1 of disease conditions and for sample size calculations.

### Native T1 in disease states

In this study, cardiac amyloidosis had distinctively high native myocardial T1, similar to previous studies [41, 51]. The distinctively low native T1 of cardiac iron overload and Anderson-Fabry disease indicate that in these diseases, the diagnosis could possibly be confirmed or ruled out using native T1-mapping in combination with other relevant imaging features and clinical information, although this needs to be tested in larger studies. For cardiac iron-overload, our patients had a less diverse range of precipitating causes (beta-thalassaemia, hereditary haemochromatosis, transfusion-dependent myelodysplasia and sickle cell anaemia) compared to those reported previously [28] which may explain the slightly lower observed T1 in our patients. For Anderson-Fabry disease, our patients had similar native T1 values compared to previous reports [39]. In patients with acute and previous myocarditis, native T1 was elevated in LGE+ myocardium, which is consistent with existing evidence of typically increased T1 in areas of acute myocyte necrosis and chronic scarring [24, 25, 27, 29, 31, 35, 52]. For chronic myocardial infarctions, we reported lower native T1 values than previously published [27], which may be related to lipomatous metaplasia. Lipomatous metaplasia increases the fat fraction within the chronic myocardial infarction, which leads to a bias in T1 estimations through the partial volume effect [51]. Kellman et al. showed that infarcts with low fat fractions (<10%) have a modest increase in T1 (~50 ms) compared to remote myocardium [51]. However, higher fat fractions (>10%) can lead to nearly 400 ms increase in the infarct T1 values, compared to remote myocardium. This effect was even greater in the infarct core with high fat fraction (35–50%) where the T1 values were ~1000 ms greater than the remote myocardium. However, this effect may disappear with extensive fatty infiltration, where theoretically the entire voxel becomes occupied by fat, and the infarct T1 in the areas of lipomatous metaplasia becomes low [53, 54]. Therefore, the differences in infarct T1 values may be accounted for by different degrees of lipomatous metaplasia present in the infarcts.

### Native T1-mapping – The quantitative cardiovascular biomarker

A fundamental limitation of LGE is that it is relatively insensitive for the detection of global pathologies, such as diffuse myocardial fibrosis, or very early changes that may not yet be apparent on LGE. In these diseases, native T1-mapping offers unique evaluation of the myocardial tissue by directly quantifying deviation from established norms. This can then detect more extensive areas of myocardial involvement even in areas without apparent LGE or any other abnormality using conventional imaging features, and may have prognostic implications in certain diseases [41, 55, 56]. In many cardiovascular diseases, once structural changes such as fibrosis set in, therapeutic options may begin to lose their effectiveness [9]. T1-mapping may enable detection of early pathological processes, and serve as a tool for early diagnosis or screening, differentiation of cardiomyopathies from normal phenotypes (e.g athletic hearts [57]), and monitoring disease progression or therapeutic response to novel treatments in clinical trials.

### Native T1 for definitive sample size calculations for future studies

For diseases with significantly higher or lower native T1 compared to controls, only a small sample size is required to detect statistically significant changes. Sample size calculations have been previously attempted, with 7 patients needed to detect 40 ms difference between samples [58]. Our large cohort provides confirmation of this, which will pave the way for tissue characterization studies and potentially clinical trials using native T1 as a safe, reproducible and gadolinium-free study endpoint.

### The role of advanced T1-map analysis and sample size calculations

Apart from cardiac amyloidosis, iron overload and Anderson-Fabry disease, native T1 mapping using basic image analysis techniques, such as septal ROI placement [2, 6–8, 29], alone cannot reliably distinguish between the remaining cardiovascular diseases, since the effect size for the T1 differences between these diseases are relatively small. Advanced image analysis techniques based on native T1 thresholds and that take into account the extent, distribution and patterns of myocardial involvement may better distinguish between different diseases or even disease spectrum within a single condition [23, 24, 32, 35].

### Limitations and future directions

This is a single-centred study addressing one specific T1-mapping technique [18], typically as a single-slice add-on to an already busy clinical exam, and rarer diseases deserve further investigation in future dedicated studies with larger sample sizes. The T1 values presented in this study were derived using a single T1-mapping technique. Due to the intrinsic technical differences, caution should be applied before directly translating values derived in this study to other T1 mapping techniques. Moreover, even within the same T1-mapping technique, different versions of sequences can lead to small differences in T1-estimations. These differences may not be immediately apparent to the operator or new users. The standardization of normative T1 values across sequences/vendors is highly desirable, but has not yet been achieved. LGE was needed in most cases to determine the location of focal abnormalities and derive myocardial T1 ranges. The T1 ranges reported in this study need to be validated in future studies for their potential standalone diagnostic value or their incremental diagnostic value to LGE. For diseases with regional abnormalities, such as CAD and HCM, the mean native T1 values are heavily dependent on the size and location of the ROI, which continues to be a limitation of all similar studies using this methodology to derive T1 values. Future development of automated image analysis techniques may enable visual diagnosis and distinction of cardiovascular diseases without the need for gadolinium-based contrast agents and serve as effective imaging endpoints for clinical trials. Finally, all sample size calculations were based on T1 values derived by ROI placement on a single mid-ventricular slice, and advanced image analysis or whole-heart coverage may decrease sample size required for clinical studies looking at diseases with small effect sizes.

## Conclusions

We provide typical T1-ranges for common clinical cardiac conditions in the largest cohort to-date, using ShMOLLI T1-mapping at 1.5 T. Sample-size calculations from this study may be useful for the design of future studies and trials that use T1-mapping as an endpoint.

## Additional files

Additional file 1: **Table S1.** Clinical indications in patients with normal CMR, but were excluded due to history of cardiovascular diseases, abnormal ECG or unclear diagnosis. (DOCX 14 kb)

Additional file 2: **Table S2.** Clinical indications in patients with normal CMR included in the study, with no history of cardiovascular diseases and had normal ECG. (DOCX 13 kb)

## Abbreviations

AF: Atrial fibrillation
AFD: Anderson Fabry disease
AS: Aortic Stenosis
BMI: Body mass index
BPM: Beats per minute
CAD: Coronary artery disease
CM: Cardiomyopathy
CMR: Cardiovascular magnetic resonance
CVD: Cardiovascular disease
DCM: Dilated cardiomyopathy
ECG: Electrocardiogram
EDV: End diastolic volume index
ESVi: End systolic volume index
GBCA: Gadolinium-based contrast agents
HCM: Hypertrophic cardiomyopathy
HR: Heart rate
HTN: Hypertension
Kg: Kilogram
LGE: Late gadolinium enhancement
LV: Left ventricle / left ventricular
LVEF: Left ventricular ejection fraction
M: Meter
Max: Maximal
Min: Minimal
Mm: Millimetre
Ms.: Millisecond
Pheo: Pheochromocytoma
ROI: Region of interest
RWMA: Regional wall motion abnormality
SD: Standard Deviation
ShMOLLI: Shortened modified look-locker inversion recovery
