# Supplementary material for: Measurement of myocardial native T1 in cardiovascular diseases and norm in 1291 subjects
Source: J Cardiovasc Magn Reson. 2017 Sep 28;19:74. doi: 10.1186/s12968-017-0386-y (PMC5618724; doi:10.1186/s12968-017-0386-y)
Supplement: Supplementary file 1 — Table S1. Clinical indications in patients with normal CMR, but were excluded due to history of cardiovascular diseases, abnormal ECG or unclear diagnosis. (DOCX 14 kb) [file 12968_2017_386_MOESM1_ESM.docx]

**Additional file 1: Table S1. Clinical indications in patients with normal CMR, but were excluded due to history of cardiovascular diseases, abnormal ECG or unclear diagnosis.**

| **Patients excluded from the study (n=53)** |  |
| --- | --- |
| Possible diagnosis of Hypertrophic Cardiomyopathy – referred for a repeat scan | 3 |
| Previous Chemotherapy | 4 |
| Systemic sarcoidosis | 3 |
| Assessment for inducible ischaemia | 6 |
| *Palpitations or bradycardia* |  |
| 1^st^ degree heart block on electrocardiogram | 2 |
| History of atrial fibrillation | 4 |
| History of supraventricular tachycardia | 5 |
| History of ventricular ectopics | 4 |
| *Investigation of ECG changes* |  |
| Abnormal R wave progression | 3 |
| T wave abnormalities | 5 |
| Bundle branch block | 2 |
| Abnormal PR interval | 4 |
| Abnormal QT interval | 3 |
| Out of Hospital cardiac arrest | 3 |
| Systolic dysfunction on echocardiography | 2 |
