# Supplementary material for: Measurement of myocardial native T1 in cardiovascular diseases and norm in 1291 subjects
Source: J Cardiovasc Magn Reson. 2017 Sep 28;19:74. doi: 10.1186/s12968-017-0386-y (PMC5618724; doi:10.1186/s12968-017-0386-y)
Supplement: Supplementary file 2 — Table S2. Clinical indications in patients with normal CMR included in the study, with no history of cardiovascular diseases and had normal ECG. (DOCX 13 kb) [file 12968_2017_386_MOESM2_ESM.docx]

**Additional file 2: Table S2. Clinical indications in patients with normal CMR included in the study, with no history of cardiovascular diseases and had normal ECG.**

| **Reason for referral** | **Number of referrals (n)** |
| --- | --- |
| **Patients included in the study** | **70** |
| Occupational screening (military and aviation) | 2 |
| Syncope with normal electrocardiogram | 10 |
| Palpitations with normal electrocardiogram | 15 |
| *Family Screening (Asymptomatic)* |  |
| Hypertrophic Cardiomyopathy | 10 |
| Arrhythmogenic Right Ventricular Cardiomyopathy | 13 |
| Dilated Cardiomyopathy | 6 |
| Non-specified “cardiomyopathy” | 8 |
| Atypical chest pain, normal ECG/cardiac enzymes | 6 |
